# Zeimssen’s Handbook of General Therapeutics

**Published:** 1885-08

**Authors:** 


### BIBLIOGRAPHY.

### BOOKS AND PAMPHLETS RECEIVED.

*Handbook of General Therapeutics*: By Prof. H. VonZiemssen, in Seven Volumes. Vol. 1.—Introduction by Prof. Von Ziemssen. Two Chapters. 1st. THE DIETARY OF THE SICK AND DIETETIC METHODS OF TREATMENT, by Prof. J. Bauer; 2nd. THE KOUMISS CURE, by Dr. Strange. Wm. Wood & Co., New York, 1885, 408 pages.

This is the first volume of the first American edition of a translation from the German, by distinguished authors, of Von Ziemssens great work on General and Special Therapeutics, (to appear in seven volumes.) Its advent has been anxiously lookfor by the English-speaking portion of the profession since its translation was undertaken.

The scope of the entire work is extensive, and yet the plan has been fully carried out, exhausting the subject. This work, which the author says was originally intended to form a constituent part of his work of Special Therapeutics, is a separate treatise, a companion to, instead of a part of the work on Special Therapeutics. "Each part, written by a German authority, eminent in his own department of practice, forms a treatise, complete in itself, dealing with the rationale and the application of the Therapeutic agents or methods which form the subject matter," says the preface. We have the first volume only. The others are in press, and soon to appear.

The author begins with a discussion of the different kinds of food and their nutritive value, (these tables are alone worth the price of the book), and then proceeds to give the kind and quantity and quality of diet suitable to certain pathological conditions. The preparation and cooking of the several kinds of food is then discussed; alchohol and other stimulants, tea, coffee, etc., are treated of, and their indications and uses in disease. The digestion of foods in health is then considered; and the effects of fever on the digestive process; the influence or effects of certain foods on the febrile state; the diet best suited to the convalescent state; the diet in diseases of the digestive organs themselves; artificial feeding; the dietetic treatment of gout, rheumatism, diabetes, anæmia and hydræmia, and finally of obesity, are all treated in a masterly manner, and with that thorough attention to the minutest detail which is so characteristic of German writers. The author then discusses the subject of vegetarianism; milk and whey cures, and their value, and this volume closes with an elaborate treatise on the Koumiss cure by Dr. Strange.

The reader has here, in this one volume, a repast as varied as it is delicious. Consisting of the most interesting and important subjects which enter into consideration in treating any and all kinds of sickness, served in a most pleasing and acceptable style, and in such form as to be mentally digested with ease and profit. This one volume is a library in itself, at once a work on physiology, pathology, materia medica and Therapeutics, with Mrs. Hale's cookery book thrown in. The work, when completed, certainly will be a library if the whole subject is treated with the elaborateness which characterizes volume one.

As to the mechanical execution of the work it is only necessary to say it is published by Wm. Wood & Co., who seem to have taken extra pains to have it reflect credit on them. The typography is perfect; the press work, faultless, and the paper, on which it is printed, is the heaviest and best sized and super-calendered paper made. We can commend the book to the practitioners (it is written for "educated and scientific men," says the author) as well worthy of a place on their book-shelf, and of a careful study; it will amply repay many hours of close study, and will certainly be a great help in every day practice.

